# Chemical composition, antioxidant capacity, and mineral extractability of Sudanese date palm (*Phoenix dactylifera* L.) fruits

**DOI:** 10.1002/fsn3.123

**Published:** 2014-05-26

**Authors:** Rania M A Mohamed, Aisha S M Fageer, Mohamed M Eltayeb, Isam A Mohamed Ahmed

**Affiliations:** Department of Food Science and Technology, Faculty of Agriculture, University of KhartoumShambat, 13314, Sudan

**Keywords:** Antioxidant, Barakawi, Bittamoda, date palm, Gondila, mineral bioavailability

## Abstract

The aim of the present work was to investigate the chemical composition, mineral extractability, and antioxidant capacity of six date palm varieties grown in Sudan. The results showed that Sudanese date varieties contained significantly different (*P* < 0.05) amounts of moisture, ash, fiber, oil, and carbohydrates, but have almost similar amounts of protein. Moreover, results revealed that date varieties contained significantly varied (*P* < 0.05) amounts of total polyphenols and total flavonoids, which ranged between 35.82 and 99.34 mg gallic acid equivalent/100 g and 1.74–3.39 mg catechin equivalent/100 g, respectively. The antioxidant activities of the studied date varieties were as follows: ferric-reducing antioxidant power (FRAP) was within the range of 2.82–27.5 mmol/100 g, chelation of Fe^2+^ ion ranged from 54.31% to 94.98%, and scavenging of H_2_O_2_ ranged from 38.48% to 49.13%. There were many correlations (positive, negative, and weak) between antioxidant and mineral extractability of Sudanese date fruits.

## Introduction

The date palm (*Phoenix dactylifera* L.) plays an important social, environmental, and economic role for many people living in arid and semiarid regions of the world. Fruits of the date palm are very commonly consumed in many parts of the world and considered as a vital component of the diet and a staple food in most Arab countries (Al-Farsi and Lee 2008). It may be one of the oldest cultivated plants, with a history of more than 6000 years. The world production of dates has increased from about 4.6 million tons in 1994 to 7.68 million tons in 2010, with expectations of continuous increase (Al-Farsi and Lee 2008). Nearly 2000 cultivars of date palm are known in the world, but only some have been evaluated for their performance and fruit quality. Dates are rich in certain nutrients and provide a good source of rapid energy, due to their high carbohydrate content (70–80%). Moreover, date fruits contain fat (0.20–0.50%), protein (2.30–5.60%), dietary fiber (6.40–11.50%), minerals (0.10–916 mg/100 g dry weight), and vitamins (C, B1, B2, B3, and A) with very little or no starch (Al-Shahib and Marshal 2003). Date fruit is also a good source of important phytochemicals, including carotenoids, phenolics, and flavonoids. Date fruit can not only provide antioxidant, antimutagenic, and immunomodulatory benefits to health but also has diverse medicinal values, including antihyperlipidemic, anticancer, gastroprotective, hepatoprotective, and nephroprotective properties (Tang et al. 2013).

In Sudan, the date palm is the most important fruit tree in the northern part of the country. It has been cultivated there for more than 3000 years, with an estimate of about 400 current varieties and strains (Osman 2001). The total number of date palm trees in northern Sudan has been estimated to be within the range of five to six million, grown in an area of about 36204 ha. Date production in the Sudan reached about 119,048 metric tons of fruit in 2010, accounting for about 5.5% of total world production (FAOSTAT 2010). Date palms contribute to the livelihoods of people in northern Sudan, as well as playing an important role in the cultural heritage of the local population. It is the most important agricultural crop in the area and provides food and income to a majority of the inhabitants. It ranks first among all crops due to its high nutritional and economic value. The annual income gained from dates is estimated to be around $200 million in the Northern and River Nile States, representing not less than 26% and 20%, respectively, of total agricultural income (Osman 2001). As by-products, wood is made from the stems, and fronds are widely used for thatching, buildings, braiding, and basketry (household utensils). Although dates have a great importance for the people of Sudan, there have been few studies on the nutritional quality (Sulieman et al. 2012) and functional properties of Sudanese dates.

In the past decade, there has been a growing interest in the chemotherapeutic and preservative properties of natural plant antioxidants to prevent oxidative reactions in food, cosmetics, and in biological systems (Molyneux 2004). Regular consumption of bioactive compounds from plants and fruit may be associated with protection against oxidative damage and lowered risk of chronic diseases, such as cancer, heart disease, and cerebrovascular disease (Hung et al. 2004). Polyphenols and flavonoids are of considerable interest to scientists, manufacturers and consumers due to their antioxidant properties (Haminiuk et al. 2012; Barbosa-Pereira et al. 2013). Although, polyphenolics and flavonoids constitute an important class of secondary metabolites that act as free radical scavengers and inhibitors of low-density lipoprotein, of cholesterol oxidation and of DNA breakage, they can also form complex with minerals and hence, reduce mineral bioavailability (Galleano et al. 2010; Rehecho et al. 2011). Thus, to understand the positive and negative effects of antioxidants on mineral bioavailability, studies on the correlation between the antioxidant capacity and mineral extractability are of great importance.

Despite the large amount of information available on the antioxidant properties and phenolic compounds of dates from various countries (Mansouri et al. 2005; Al-Farsi et al. 2007; Biglari et al. 2008; Benmeddour et al. 2013), information regarding the antioxidant potential of Sudanese dates is scarce. To the best of our knowledge, limited data are available on the chemical composition of dates grown in Sudan (Sulieman et al. 2012). To date, there has been no data published on the antioxidant capacity, polyphenols, flavonoids, and mineral extractability of Sudanese date fruits. Detailed information on the nutritional composition and health-promoting components of dates will enhance our knowledge and appreciation for the use of dates and their products in a variety of food and specialty products, including their use as functional foods and ingredients in nutraceuticals, pharmaceuticals, and medicine. Given this, the objectives of the present research were to investigate the chemical composition, mineral content and extractability, and antioxidant capacity of Sudanese date fruits.

## Materials and Methods

### Materials

The dates acquired were Barakawi, Gondaila, Jaw, Mishrig, Bittamoda, and Madini. These six varieties are the most common varieties locally grown and utilized in Sudan. The fruits of these varieties were purchased from a well-known date's market in Khartoum State, Khartoum, Sudan, at the beginning of the 2010 harvest season. The origin of these fruits is a date farm in Dongola region, northern Sudan. They had been subjected to uniform harvest, postharvest, and handling practices. In these processes, the fruits were harvested manually on a clean plastic sheets, sun dried, packed, and transported to the market. The date fruits selected and used were of uniform size and free of physical damage, insects, injury, and fungal infection. Samples were manually cleaned, pitted, and blended with a blender and kept in polyethylene bags at 4°C for further investigations. The experiment was designed as a completely randomized design with three replicates. Ten dates were used for each replication for each type of date. Unless otherwise stated, all chemicals used in the study were of analytical grade.

### Approximate analysis

The determination of moisture, crude fiber, crude fat, crude protein, and ash were carried out according to the official standard method (AOAC 2003). The total carbohydrate of the samples was calculated by subtracting the value of protein, oil, fiber, ash, and moisture content from 100.

### Determination of minerals content and extractability

Minerals content were determined by the dry ashing method (Chapman and Pratt 1982). Calcium and magnesium (Mg) were measured by titration. Phosphorus was determined spectrophotometrically using the molybdovanadate method. All other minerals were determined by atomic absorption spectrophotometer (Shimadzu AA-680, Shimadzu, Japan).

HCl extractability of minerals (in vitro bioavailability) was determined by the method of Chauhan and Mahjan (1988). Briefly, 1 g of the sample was shaken with 10 mL of 0.03 mol/L HCl for 3 h at 37°C and then filtered. The clear extract obtained was oven dried at 100°C and then acid digested. The amount of extractable minerals was determined by the above described methods. HCl extractability of minerals (%) was determined as follows:


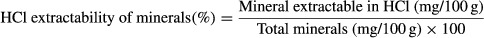


### Extraction

One hundred grams of the edible part of the date palm fruit was extracted with 300 mL methanol–water (4:1, v/v) at room temperature (20°C) for 5 h using an orbital shaker. The extracts were then filtered and centrifuged (Hettich Zentrifugen, Tuttlingen, Germany) at 4000*g* for 10 min. The supernatant was concentrated under reduced pressure at 40°C for 3 h using a rotary evaporator (IKA-WERKE-RV06ML; Staufen, Germany) to obtain the date palm fruit methanolic crude extract. The crude extract was kept in dark glass bottles inside the freezer until use. The storage conditions (time and temperature) were the same for all types of fruit.

### Determination of polyphenols

Total polyphenols were determined as described by Al-Farsi et al. (2005a). The results were expressed as milligram gallic acid equivalents per 100 g of dry weight (mg GAE/100 g DW).

### Determination of total flavonoids content

Total flavonoids content (TFC) of the date extracts were measured according to the colorimetric assay of Kim et al. (2003). One milliliter of the methanolic extract was added to 300 *μ*L sodium nitrite solution (5%) followed by 300 *μ*L aluminum chloride (10%). Test tubes were incubated at room temperature for 5 min, and then 2 mL of 1 mol/L sodium hydroxide was added. Immediately, the volume of reaction mixture was made to 10 mL with distilled water and the mixture was thoroughly vortexed. The absorbance of the mixture was determined at 510 nm. Total flavonoid content was reported as milligrams of catechin equivalents per 100 g (mg CE/100 g DW).

### Determination of antioxidant capacities

#### Ferric-reducing antioxidant power

The FRAP of samples was determined according to the method described by Oyaizu (1986). A stock solution of each date variety in methanol (1 mg/mL) was prepared and different volumes (125, 250, 500, and 1000 *μ*L) from each stock solution were transferred to different test tubes. The volume in each test tube was adjusted to 1 mL with the same solvent. Then, 2.5 mL of 200 mmol/L sodium phosphate buffer (pH 6.6), and 2.5 mL of 1% potassium ferricyanide were added to each test tube and incubated at 50°C for 20 min. After incubation, 2.5 mL of 10% trichloroacetic acid was added and centrifuged at 2000*g* for 10 min. The upper layer (2.5 mL) was mixed with 2.5 mL of deionized water and 0.5 mL of 0.1% ferric chloride. The absorbance was measured at 700 nm against a blank. The FRAP of each date sample at different concentrations was compared to ascorbic acid as a positive control and the results were expressed as ascorbic acid equivalent.

#### Chelation of Fe^2+^ ions

Concentration of free iron ions (Fe^2+^) was estimated using chelating agent 2,2-dipyridyl as described by Harris and Livingstone (1964). Briefly, a stock solution of each date variety containing 1 mg/mL in methanol was prepared and different amounts (125, 250, 500, and 1000 *μ*L) from each stock solution were transferred to different test tubes. The volume in each test tube was adjusted to 1 mL with the same solvent. To each tube, 1 mL of a solution containing 50 mmol/L FeSO_4_ and 50 mmol/L NaCl (pH 7.0) was added. A blank solution was prepared using 1 mL of methanol instead of the sample. Samples were incubated for 30 min at room temperature and then 2 mL of 2,2-dipyridyl (1 mmol/L) was added. Absorbance of ferrous–dipyridyl complex was measured at 525 nm against a solution devoid of ferrous sulfate. The results were expressed as a percentage of inhibition of 2,2-dipyridyl–Fe^2+^ complex formations.

#### Hydrogen peroxide scavenging capacity

The hydrogen peroxide (H_2_O_2_) scavenging ability of palm dates was measured using the method described by Jayaprakasha et al. (2004). A solution of H_2_O_2_ (40 mmol/L) was prepared in phosphate buffer (pH 7.4). Various concentrations (125, 250, 500, and 1000 *μ*L) of date extract were prepared in 40 mmol/L phosphate buffer saline (pH 7.4). Then, 1 mL of H_2_O_2_ solution (40 mmol/L) was added and the reaction mixtures were incubated for 10 min at room temperature. Absorbance of H_2_O_2_ at 230 nm was determined after 10 min against a blank solution containing phosphate buffer without hydrogen peroxide. The scavenging capacity was calculated using the following formula:


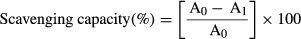


where A_0_ is the absorbance of the control and A_1_ is the absorbance of the sample extracts.

### Statistical analysis

For all the experiments, three samples of each date were analyzed and the entire assay was carried out in triplicate. Results were analyzed using one way analysis of variance (ANOVA) and Tukey's multiple comparison tests were used to compare between treatment means. The significance level was accepted at *P* < 0.05. The correlation between antioxidant and mineral extractability was assessed using the Pearson criteria (*P* < 0.05 and 0.01) using the Statistical Analysis System (SAS, v. 8.1; SAS Institute Inc., Cary, NC).

## Results and Discussion

### Approximate composition

The chemical composition of the six Sudanese date varieties studied is shown in Table 1. With the exception of protein, ANOVA showed significant (*P* < 0.05) varietal differences of chemical components in all Sudanese date fruits. The moisture, ash, fiber, protein, fat, and carbohydrate content were within the ranges of 8.78–10.68, 1.96–2.50, 2.37–3.14, 1.71–2.06, and 78.73–80.41 g/100 g, respectively. These results were in general agreement with those reported previously (Ahmed et al. 1995; Al-Farsi et al. 2007; Amira et al. 2011; Baliga et al. 2011; Tang et al. 2013). However, in contrast, different amounts of these chemical components in dates from various countries have also been reported (Al-Shahib and Marshal 2003; Al-Farsi et al. 2005a). These variations could be attributed to differences in cultivar, harvest/postharvest practices, and growing environment such as soil fertility, temperature, humidity, etc. Our data revealed that Sudanese date fruits contained sufficient amounts of most essential nutrients, and thus could be recommended for regular consumption. The low level of lipid content compared with the high sugar content of dates is a good indicator for its potential uses. Interestingly, date fruits are usually characterized by high carbohydrate content (up to 88%), most of which is in the form of digestible sugar such as glucose, fructose, and sucrose (Baliga et al. 2011). Because of this, sugars in dates are the most important constituent as they provide a rich source of energy to the human system. Approximately 100 g of flesh can provide 314 kcal of energy (Al-Farsi et al. 2005a; Al-Farsi and Lee 2008). This could also elevate blood sugar after rapid digestion of reducing sugars such as glucose. Beside their value as an energy source, date sugars are also used as sweeteners of foods especially in the preparation of beers (Al-Farsi et al. 2005a; Al-Farsi and Lee 2008). Although the sugar types of Sudanese dates were not investigated in this study, it can be assumed from the higher concentration of total carbohydrate and/or appreciable amounts of fiber and protein that Sudanese date fruits have good nutritional and health potential.

**Table 1:** Chemical composition (g/100 g) of different Sudanese date varieties

Varieties	Moisture	Ash	Fiber	Protein	Fat	Carbohydrate
Gondaila	8.78 ± 0.10^d^	2.20 ± 0.03^c^	2.53 ± 0.12^b^	4.09 ± 0.15^a^	2.00 ± 0.08^ab^	80.41 ± 1.01^a^
Barakawi	9.38 ± 0.09^c^	2.50 ± 0.13^a^	2.67 ± 0.09^b^	4.03 ± 0.12^a^	1.87 ± 0.05^bc^	79.55 ± 1.03^ab^
Jaw	10.68 ± 0.19^a^	2.38 ± 0.11^ab^	2.74 ± 0.13^ab^	3.69 ± 0.06^b^	1.79 ± 0.04^cd^	78.73 ± 0.71^b^
Mishrig	8.81 ± 0.07^d^	2.19 ± 0.05^c^	2.78 ± 0.19^ab^	3.92 ± 0.10^ab^	2.06 ± 0.05^a^	80.27 ± 0.27^a^
Bittamoda	10.03 ± 0.13^b^	1.96 ± 0.09^d^	2.37 ± 0.16^b^	3.72 ± 0.09^ab^	1.81 ± 0.03^c^	80.11 ± 0.44^a^
Madini	9.90 ± 0.12^b^	2.27 ± 0.05^b^	3.14 ± 0.11^a^	3.94 ± 0.04^a^	1.71 ± 0.01^d^	79.04 ± 0.43^b^

Means (±SD, *n* = 3) in the same column sharing the same letter(s) are not significantly different at *P* < 0.05.

### Total and extractable macrominerals

Significant varietal (*P* < 0.05) differences existed in macromineral composition and extractability of Sudanese date varieties (Table 2). The calcium content of different date varieties was within a range of 222.2–293.04 mg/100 g and was significantly (*P* < 0.05) different between date varieties. These findings were within the wide range 140–385 mg/100 g reported for some Iranian dates at the Tamr stage (Rastegar et al. 2012). However, our results were much higher than those reported by many investigators (Ahmed et al. 1995; Al-Shahib and Marshal 2003; Al-Farsi et al. 2005a; Baliga et al. 2011; Tang et al. 2013). Despite the high calcium content of Sudanese date varieties, only 20–37.5% of the total calcium was extractable. Madini cultivar has shown the highest extractability of calcium, whereas Bittamoda has the lowest. Mineral extractability of Sudanese date cultivars varied significantly (*P* < 0.05). Barakawi exhibited high calcium content and a good degree of calcium extractability. Although many studies have described the mineral contents of date fruits from various origins, none of them have looked at the extractability of these minerals. To the best of our knowledge, this is the first report on mineral bioavailability of date fruits.

**Table 2:** Total (mg/100 g) and extractability (%) of macrominerals of different Sudanese date varieties

	Ca		Mg		Na		K		P	
Varieties	mg/100 g	%	mg/100 g	%	mg/100 g	%	mg/100 g	%	mg/100 g	%
Gondaila	238.10 ± 12.63^b^	25.98 ± 1.85^b^	120.88 ± 7.78^a^	73.17 ± 2.05^a^	131.87 ± 1.01^c^	9.82 ± 0.70^c^	862.64 ± 7.98^c^	7.89 ± 0.55^b^	208.79 ± 4.06^b^	24.39 ± 0.75^d^
Barakawi	276.24 ± 10.08^a^	32.93 ± 1.99^ab^	66.30 ± 2.67^c^	41.10 ± 1.87^c^	135.36 ± 0.76^b^	11.09 ± 1.31^c^	1088.40 ± 13.84^a^	4.81 ± 0.05^f^	232.04 ± 3.78^a^	33.79 ± 0.30^b^
Jaw	223.46 ± 9.18^b^	33.76 ± 2.04^ab^	111.73 ± 6.55^a^	46.42 ± 5.00^bc^	139.11 ± 1.18^a^	8.81 ± 0.81^c^	974.86 ± 5.45^b^	6.82 ± 0.24^c^	212.29 ± 5.11^b^	20.66 ± 1.05^e^
Mishrig	293.04 ± 15.21^a^	24.39 ± 1.15^b^	109.89 ± 8.92^a^	31.17 ± 0.04^d^	60.77 ± 0.22^d^	27.16 ± 0.98^a^	733.52 ± 4.16^d^	6.30 ± 0.45^d^	150.19 ± 5.70^d^	27.67 ± 0.43^c^
Bittamoda	277.78 ± 9.08^a^	20.00 ± 1.05^c^	100.00 ± 5.08^b^	55.50 ± 4.52^b^	55.56 ± 0.42^f^	22.50 ± 1.05^b^	722.56 ± 5.10^e^	8.59 ± 0.43^a^	166.67 ± 3.58^c^	25.84 ± 0.97^d^
Madini	222.20 ± 6.22^b^	37.50 ± 3.45^a^	66.67 ± 4.18^c^	50.00 ± 3.15^b^	56.67 ± 0.30^e^	26.19 ± 0.65^a^	691.67 ± 9.08^f^	5.18 ± 0.15^e^	170.33 ± 2.00^c^	42.44 ± 1.65^a^

Means (±SD, *n* = 3) in the same column sharing the same letter(s) are not significantly different at *P* < 0.05.

The Mg content of Sudanese date types ranged from 66.3 to 120.88 mg/100 g (Table 2). The Gondaila variety had the highest value, whereas Madini and Barakawi had the lowest Mg content. These results were, in general, comparable with the ranges reported for dates from different origins, such as 60.9–76.2 mg/100 g (Al-Farsi et al. 2005a) and 47–82 mg/100 g (Ahmed et al. 1995; Al-Shahib and Marshal 2003). The findings were also within the range 31.0–150 mg/100 g (Baliga et al. 2011), and slightly lower than the range 114–250 mg/100 g reported for Iranian dates (Rastegar et al. 2012; Tang et al. 2013). These differences in Mg content between these studies could be due to the varieties, environmental conditions, soil fertility, and agronomic practices. Mg extractability of date cultivars was significantly (*P* < 0.05) different and showed a very wide range from 31.17% to 73.17%. The Gondaila variety showed the highest percentage of Mg extractability, whereas Mishrig showed the lowest, although it had a good amount of Mg (109.89 mg/100 g). Interestingly, more than 50% of total Mg content of the varieties Madini, Bittamoda, and Gondaila are available for absorption in the digestive track of the human body.

The sodium (Na) content of Sudanese date cultivars varied significantly (*P* < 0.05) and were in the range 55.56–139.11 mg/100 g with the highest value in Jaw and the lowest in Bittamoda (Table 2). These values are in general agreement with previously published studies on various date varieties (Ahmed et al. 1995; Al-Shahib and Marshal 2003; Baliga et al. 2011; Rastegar et al. 2012; Tang et al. 2013). Our results also showed higher Na content than those of other investigators (Al-Farsi et al. 2005a; Ismail et al. 2006). Sodium bioavailability of Sudanese date varieties were significantly different (*P* < 0.05) and were within the range of 8.81–27.16%. Sodium bioavailability of date cultivars was very low. This could be an interest finding especially for people with hypertension problems.

Significant (*P* < 0.05) varietal differences were observed in the potassium content of Sudanese date varieties. Among the minerals studied, the potassium content of Sudanese dates was most abundant with the concentration of 691.67–1088.4 mg/100 g (Table 2). Barakawi cultivar had the highest potassium concentration, while Madini had the lowest. Interestingly, all the varieties were high in potassium concentration but low in sodium concentration (55.56–139.11 mg/100 g). The high potassium–sodium ratio potentially makes the date fruit a desirable food for people suffering from hypertension. Our results were similar to various ranges reported for dates from different varieties such as 345–1287 mg/100 g (Baliga et al. 2011), 524–1164 mg/100 g (Ismail et al. 2006), 565–916 mg/100 g (Ahmed et al. 1995), 107.4–916 mg/100 g (Al-Shahib and Marshal 2003), and 603–742 mg/100 g (Al-Farsi et al. 2005a). A high amount of potassium in date fruits was also reported by several researchers (Rastegar et al. 2012; Tang et al. 2013). Despite the higher amount of potassium found in date fruits in this study, the bioavailability of this element was very low. Only 4.81–8.59% of the total potassium of date cultivars studied was available for absorption. This could be attributed to the fact that the presence of polyphenols is usually associated with lower extractability of minerals. Before making assumptions about the nutritional benefits of high potassium values in date fruits, however, there is a need to study the mineral extractability aside from the mineral content of date fruits.

The phosphorus content of the date cultivars was significantly different (*P* < 0.05) and was within the range of 150.19–232.04 mg/100 g (Table 2). The highest phosphorus content was found in Barakawi, with the lowest in Mishrig. As compared to previous reports on the phosphorus content of dates from different countries (Al-Shahib and Marshal 2003; Al-Farsi et al. 2005a; Baliga et al. 2011), Sudanese date varieties were found to have a higher phosphorous content. This could be due to variations in date genotypes, cultivation practices, ripening stage, soil fertility, and environmental conditions. On the other hand, an extractability study indicated that only 20.66–42.44% from the total phosphorus of Sudanese date varieties studied was available for absorption. Phosphorus extractability of date cultivars varies significantly (*P* < 0.05) between varieties, in which Madini shows the highest percentage of extractability and Jaw the lowest. Collectively, Sudanese date varieties demonstrated a high amount of macromineral content with a significant varietal dependence. The higher concentration of these nutrients was only hindered by their somewhat lower extractability. This lower extractability is attributed to the presence of antinutritional factors such as polyphenols and flavonoids in date fruits.

### Total and extractable microminerals

Micronutrients are involved in a high numbers of biological processes, as a component of proteins or as essential components of numerous enzymes required for oxidative, amino acids, lipids or carbohydrate metabolism. The results presented in Table 3 showed significant varietal (*P* < 0.05) differences in micromineral content and extractability. The concentration of iron (Fe) in Sudanese dates ranged from 4.06 mg/100 g in Barakawi to 7.06 mg/100 g in Jaw. ANOVA showed a significant (*P* < 0.05) difference in Fe content between all date varieties. Our results on Fe content fell within the range of 0.3–10.4 mg/100 g reported previously for date varieties from different countries (Al-Shahib and Marshal 2003). The current findings were higher than the range 0.3–1.5 mg/100 g (Ahmed et al. 1995), 0.58–1.09 mg/100 g (Al-Farsi et al. 2005a), 0.1–1.5 mg/100 g (Baliga et al. 2011), 0.83–1.76 mg/100 g (Ismail et al. 2006), and 1.6–1.8 mg/100 g (Rastegar et al. 2012; Tang et al. 2013) reported for dates from various countries. On the other hand, Fe extractability of Sudanese date fruits varied significantly (*P* < 0.05) and ranged between 8.67% and 37.0%. Although the variety Barakawi had the lowest Fe content it showed higher extractability. In contrast, the variety Jaw, which had the highest Fe value, showed lower extractability. This finding demonstrates that Fe content and extractability of Sudanese date fruits are variety-dependent factors.

**Table 3:** Total (mg/100 g) and extractability (%) of microminerals of different Sudanese date varieties

	Fe		Cu		Zn		Mn	
Varieties	mg/100 g	%	mg/100 g	%	mg/100 g	%	mg/100 g	%
Gondaila	6.48 ± 0.28^c^	23.04 ± 1.01^c^	0.71 ± 0.01^e^	86.93 ± 0.86^a^	0.75 ± 0.02^d^	89.34 ± 3.53^b^	0.78 ± 0.04^a^	85.89 ± 5.89^b^
Barakawi	4.06 ± 0.21^f^	37.00 ± 0.54^a^	1.27 ± 0.08^b^	44.10 ± 2.23^c^	0.72 ± 0.01^e^	72.17 ± 1.69^d^	0.59 ± 0.01^c^	100.00 ± 6.35^a^
Jaw	7.06 ± 0.10^a^	8.67 ± 0.68^e^	0.89 ± 0.06^d^	79.11 ± 0.95^b^	0.78 ± 0.00^c^	77.76 ± 3.11^c^	0.74 ± 0.02^ab^	66.81 ± 7.01^c^
Mishrig	5.50 ± 0.13^d^	17.51 ± 0.19^d^	1.04 ± 0.05^c^	87.29 ± 1.10^a^	0.66 ± 0.01^f^	99.60 ± 4.32^a^	0.71 ± 0.04^b^	45.02 ± 1.61^d^
Bittamoda	5.09 ± 0.11^e^	29.58 ± 0.40^b^	1.09 ± 0.07^c^	78.65 ± 0.37^b^	0.83 ± 0.04^b^	71.66 ± 2.45^d^	0.68 ± 0.03^b^	13.69 ± 3.02^f^
Madini	6.91 ± 0.04^b^	36.18 ± 0.83^a^	1.86 ± 0.12^a^	33.50 ± 1.03^d^	1.00 ± 0.07^a^	55.55 ± 4.17^e^	0.54 ± 0.01^d^	25.51 ± 2.23^e^

Means (±SD, *n* = 3) in the same columns sharing the same letter(s) are not significantly different at *P* < 0.05.

ANOVA showed significant (*P* < 0.05) differences in copper (Cu) content and extractability of Sudanese date varieties (Table 3). The Cu content of Sudanese dates ranged from 0.71 mg/100 g in the variety Gondaila to 1.86 mg/100 g in the cultivar Madini. These results were within the range of 0.1–2.9 mg/100 g reported for some date varieties from other countries (Al-Shahib and Marshal 2003). However, our results were higher than those reported for Cu concentration in dates from several countries (Ahmed et al. 1995; Al-Farsi et al. 2005a; Baliga et al. 2011). This variation could be due to variation in date genotypes, cultivation practices, ripening stage, and soil fertility. On the other hand, extractability of Cu varied (*P* < 0.05) between date varieties and showed a wide percentage range (33.50–87.93%). Surprisingly, more than 77% of Cu in the four date varieties (Mishrig, Gondaila, Jaw, and Bittamoda) of this study was extractable and available for absorption. In contrast, the other cultivars (Barakawi and Madini) showed more than 33% bioavailability of Cu in Sudanese dates. A higher extractability of Cu in date fruits is an indication of better utilization by the human body.

Significant (*P* < 0.05) varietal differences in zinc (Zn) concentration and extractability of six Sudanese date varieties were also evident from the results in Table 3. The concentration of Zn in Sudanese dates varied from 0.66 mg/100 g in Mishrig to 1.0 mg/100 g in Madini. In agreement with our results, Al-Shahib and Marshal (2003) reported a wide range of Zn concentration (0.1–1.8 mg/100 g) in dates from various countries. In contrast, significantly lower concentrations of Zn in date fruits from various countries was reported (Ahmed et al. 1995; Al-Farsi et al. 2005a; Ismail et al. 2006; Baliga et al. 2011). However, a higher content of Zn at the Tamr stage of ripening in three Iranian dates was recently reported (Rastegar et al. 2012; Tang et al. 2013). The variation in Zn content between these studies could be attributed to differences in varieties, agronomical practices, ripening stage, soil fertility, and environmental conditions. On the other hand, an in vitro extractability study revealed that more than 50% of the total Zn of Sudanese date varieties studied was available for absorption in the digestive tract. The varieties Mishrig (99.60%) and Gondaila (89.34%) presented the first and second highest levels of Zn extractability, respectively.

Manganese (Mn) concentrations of Sudanese date fruits ranged between 0.54 and 0.78 mg/100 g with slight significant differences between date varieties (Table 3). The highest Mn content was found in Gondaila, whereas the lowest was observed in Madini. Compared with previous reports on Mn content of date fruits from different origins, our results fell within the ranges 0.3–5.9 mg/100 g (Al-Shahib and Marshal 2003) and 0.4–1.6 mg/100 g (Rastegar et al. 2012; Tang et al. 2013). In contrast, other studies reported lower concentrations of Mn in dates from various countries (Ahmed et al. 1995; Al-Farsi et al. 2005a; Ismail et al. 2006; Baliga et al. 2011). On the other hand, ANOVA showed significant (*P* < 0.05) varietal differences in the in vitro extractability of Mn. A wide range of Mn extractability was also observed with a maximum extractability of 100% in Barakawi and a minimum extractability of 13.69% in Bittamoda. Taken together, the micromineral concentrations of Sudanese date varieties were generally higher than those reported previously for other date varieties. With the exception of iron, the extractability of all micronutrients in this study was found to be good.

Overall, the values of minerals assessed in date palm fruits were generally high compared with other fruits (Rastegar et al. 2012). It has been reported that the percentages of potassium, phosphorus, and iron in dates were much higher than in other fruits (Al-Shahib and Marshal 2003). The amount of these three minerals in dates was three to five times more than their amount in grapes, apples, oranges, and bananas (Al-Showiman 1990). With few exceptions, concentration and in vitro extractability of both macro- and microminerals of Sudanese date varieties was generally good. Thus, it can be assumed that the consumption of these dates could efficiently supply the body with the recommended dietary allowance of these minerals.

### Total polyphenol content

A comparison of total polyphenol content (TPC) data of the Sudanese date varieties tested is presented in Table 4. The TPC of different date varieties varied considerably (*P* < 0.05) and ranged from 35.82 to 199.34 mg GAE/100 g DW. The order of TPC of Sudanese date varieties was as follows: Madini > Bittamoda > Mishrig > Jaw > Gondaila > Barakawi. The results were within the range 3.91–661 mg/100 g reported for dates from different origins (Baliga et al. 2011). The results were also higher compared to those reported by Mansouri et al. (2005) who found that TPC of methanolic extract of ripe Algerian date fruits varied from 2.49 to 8.36 mg GAE/100 g FW. Furthermore, the results were slightly higher than the range 2.89–141.35 mg GAE/100 g DW reported for some Iranian soft, semidry and dry dates (Biglari et al. 2008). In contrast, higher TPC of date fruits from different countries has been reported by a number of authors (Al-Farsi et al. 2005b; Benmeddour et al. 2013). The observed differences among these studies may be related to the cultivars, environmental conditions, fruit maturity, fruit moisture content, and extraction conditions such as solvent and ratio of material/solvent (Al-Farsi et al. 2005b; Benmeddour et al. 2013). Comparative studies with fresh and dried dates have shown a significant increase in phenolic content ensures on drying, possibly due to the degradation of tannins and maturation of degradative enzymes at higher temperatures (Al-Farsi et al. 2005b). This study revealed that Sudanese date varieties have high TPC as compared to other fruits such as blueberry (46.24 mg GAE/100 g FW), mango (35 mg GAE/100 g FW), cantaloupe (31.50 mg GAE/100 g FW), peach (27.58 mg GAE/100 g FW), royal banana (25.55 mg GAE/100 g FW), green grape (23.20 mg GAE/100 g FW), avocado (21.86 mg GAE/100 g FW), olive (21.68 mg GAE/100 g FW), and pear (11.88 mg/100 g FW) (Fu et al. 2011). It can thus be assumed that Sudanese date varieties serve as a good source of polyphenolic compounds that could potentially be used in food and nutraceutical formulations.

**Table 4:** Total polyphenols content (TPC) and total flavonoids content (TFC) for different Sudanese date varieties

Varieties	TPC(mg GAE/100 g DW)	TFC(mg CE/100 g)
Gondaila	55.82 ± 3.68^e^	3.39 ± 0.09^a^
Barakawi	35.82 ± 5.01^f^	1.85 ± 0.01^c^
Jaw	63.24 ± 5.07^d^	1.81 ± 0.01^d^
Mishrig	111.65 ± 9.22^c^	1.74 ± 0.00^e^
Bittamoda	124.89 ± 7.48^b^	3.27 ± 0.07^b^
Madini	199.34 ± 9.51^a^	1.74 ± 0.04^e^

Means (±SD, *n* = 3) in the same column sharing the same letters(s) are not significantly different at *P* < 0.05.

### Total flavonoids content

The TFC of the six Sudanese date varieties varied significantly (*P* < 0.05) and ranged from 1.74 to 3.39 mg CE/100 g (Table 4). The Gondaila variety had the highest value, while Mishrig and Madini showed the lowest. The order of TFC content of Sudanese date palm fruits was as follow: Gondaila > Bittamoda > Barakawi > Jaw > Mishrig and Madini. In agreement with our results, Biglari et al. (2008) have reported that the TFC of Iranian date varieties ranged between 1.62 and 81.79 mg catechin equivalents/100 g DW. In contrast, Benmeddour et al. (2013) reported a large variation and significant (*P* < 0.05) differences in TFC among various Algerian date varieties from 15.22 to 299.74 mg QE/100 g DW. The differences in TFC between these studies could be due to the cultivars, environmental conditions, fruit maturity, fruit moisture content, and extraction conditions. It is well known that flavonoids present in plants possess diverse health benefits, which include antioxidant and radical scavenging activities, reduction in certain chronic diseases, prevention of some cardiovascular disorders, and of certain kinds of cancerous processes (Tapas et al. 2008). Although it is established that flavonoids are important phenolic compounds that contribute to the antioxidant activity of date palm fruits, it is possible that other phenolic compounds could also contribute to the antioxidant properties of these types of dates.

### Antioxidant capacity

#### Ferric-reducing antioxidant power

The FRAP significantly (*P* < 0.05) increased with increases in the concentration (125–1000 *μ*g) of Sudanese date palm fruits extracts (Table 5). The results also revealed that FRAP of all date palm fruits showed significant (*P* < 0.05) varietal differences and ranged between 2.82 and 27.50 mmol/100 g. The cultivar Bittamoda showed greater FRAP at extract concentrations of 125, 250, and 500 *μ*g/mL. In contrast, Jaw cultivar showed the highest FRAP at an extract concentration of 1000 *μ*g/mL. On the other hand, Barakawi cultivar had the lowest FRAP values at an extract concentration of 125–500 *μ*g/mL. With regard to FRAP, the cultivar Bittamoda was found superior as compared to other cultivars. These results were also constituent with those of total polyphenol and total flavonoid contents of the same date variety. In agreement with our results, previous reports on date fruits from different countries showed significant varietal differences in FRAP (Biglari et al. 2008; Al-Mamary et al. 2010; Benmeddour et al. 2013). Furthermore, similar to our observations, the FRAP of date palm fruits from different origins appeared in a dose-dependent fashion (Biglari et al. 2008; Al-Mamary et al. 2010). In addition, this study revealed that Sudanese date varieties have high FRAP as compared with many other fruits (Fu et al. 2011). The FRAP of date fruits measured by the method used in this study could be related to phenolic and polyphenolic compounds, which reduced ferricyanide ion ([Fe (CN)_6_]^3−^) to ferrocyanide ions ([Fe (CN)_6_]^4−^), which reacts with Fe^3+^ ions to give the compound called Prussian blue-colored complex (i.e., ferric ferrocyanide, Fe_4_[Fe (CN)_6_]_3_. This reduction occurs due to the electron (or ^•^H) donating ability of palm date fruits containing phenolic and polyphenolic compounds having more OH groups. These OH groups act as more powerful reducing agents, because they have more electron-donating abilities that result in the cessation of free radical chain reactions.

**Table 5:** Ferric reducing antioxidant power (FRAP), chelation of Fe^2+^ (Fe^2+^chel), and scavenging of H_2_O_2_ (SA of H_2_O_2_) at different concentrations of methanolic extracts of six Sudanese date varieties

	FRAP (mmol/100 g)				Fe^2+^chel (%)				SA of H_2_O_2_ (%)			
	Date extract concentration (*μ*g/mL)											
Varieties	125	250	500	1000	125	250	500	1000	125	250	500	1000
Gondila	3.91 ± 0.08^t^	5.44 ± 0.01^p^	10.75 ± 0.09^j^	17.60 ± 0.15^f^	71.71 ± 0.44^i^	75.39 ± 0.09^g^	76.02 ± 0.18^f^	79.31 ± 0.33^d^	40.74 ± 0.05^r^	44.91 ± 0.21^n^	47.74 ± 0.14^f^	48.25 ± 0.13^d^
Barakawi	2.82 ± 0.03^w^	5.31 ± 0.05^q^	10.16 ± 0.00^k^	19.75 ± 0.11^c^	64.73 ± 0.13^m^	65.55 ± 0.25^l^	65.83 ± 0.01^k^	73.59 ± 0.75^h^	40.70 ± 0.13^r^	45.27 ± 0.10 ^m^	48.56 ± 0.09^c^	48.87 ± 0.04^b^
Jaw	4.16 ± 0.11^s^	7.79 ± 0.14^m^	14.25 ± 0.13^g^	27.56 ± 0.09^a^	64.50 ± 0.00^n^	66.31 ± 0.17^j^	79.78 ± 0.29^c^	94.98 ± 0.24^a^	40.28 ± 0.08^s^	45.47 ± 0.08 ^l^	48.58 ± 0.10^c^	49.13 ± 0.10^a^
Mishrig	3.38 ± 0.02^v^	6.00 ± 0.11^o^	11.88 ± 0.05^i^	19.00 ± 0.08^d^	54.30 ± 0.58^w^	56.35 ± 0.37^u^	60.19 ± 0.07^r^	75.47 ± 0.22^g^	41.51 ± 0.00^q^	44.14 ± 0.01^o^	47.27 ± 0.02 ^h^	47.33 ± 0.03^g^
Bittamoda	5.06 ± 0.05^r^	8.38 ± 0.17 ^l^	13.73 ± 0.02^h^	18.25 ± 0.12^e^	55.41 ± 0.00^v^	57.52 ± 0.46^t^	59.33 ± 0.61^s^	79.94 ± 0.14^b^	43.98 ± 0.26^p^	47.02 ± 0.10^i^	48.25 ± 0.07^d^	48.87 ± 0.21^b^
Madini	3.59 ± 0.10^u^	6.94 ± 0.09^n^	13.75 ± 0.03^h^	27.25 ± 0.06^b^	60.42 ± 0.10^q^	61.29 ± 0.20^p^	61.83 ± 0.32^o^	78.76 ± 0.50^e^	38.48 ± 0.33^t^	46.19 ± 0.12^k^	46.55 ± 0.00^j^	47.94 ± 0.11^e^

Means (±SD, *n* = 3) within rows and columns sharing the same letters(s) are not significantly different at *P* < 0.05.

#### Chelation of Fe^2+^ ions

The results in Table 5 showed that all tested date fruit extracts exhibited metal-chelating activity at all concentrations. The metal-chelating activities of the Sudanese date fruit extracts were concentration dependent as evident from the increase in Fe chelating percentage with increasing concentrations (125–1000 *μ*g/mL) of date fruit extracts. At the lower extract concentration (125, 250 *μ*g/mL), the cultivar Gondaila exhibited the best chelating activity, whereas at higher concentrations (500, 1000 *μ*g/mL), the cultivar Jaw had the highest value. The chelation of Fe^2+^ ions of palm date fruits at different concentrations differed significantly (*P* < 0.05) and were within the range of 54.31–94.98%. These findings demonstrate that Sudanese date fruits have intermediate to high iron binding capacity at the tested levels, which mean that these date fruits can act as peroxidation protectors. In agreement with our results, a concentration-dependent metal chelating ability has been reported for palm date syrups (Al-Mamary et al. 2010). In addition, Benmeddour et al. (2013) have investigated the antioxidant capacity of Algerian date fruits and found that the iron chelating ability of these dates ranged between 47.6% and 95.5%. In contrast, low to intermediate values (10.26–55.42%) of iron chelating capacity for Yemeni, Saudi, and Iraqi date syrups have also been reported (Al-Mamary et al. 2010). The differences between these results could be attributed to varietal difference, ripening stage, extraction, and environmental conditions.

#### Scavenging of H_2_O_2_

The H_2_O_2_ scavenging capacity of Sudanese date fruit extracts varied significantly (*P* < 0.05) having the range 38.48–49.13%. This significantly increased with an increase in the concentration (125–1000 *μ*g/mL) of Sudanese date palm fruit extracts. The cultivar Bittamoda showed the greatest H_2_O_2_ scavenging activity at extract concentrations of 125, 250, and 500 *μ*g/mL. In contrast, the cultivar Jaw showed the highest H_2_O_2_ scavenging activity at the extract concentration of 1000 *μ*g/mL. In this regard, the cultivar Bittamoda is superior in H_2_O_2_ scavenging capacity as compared to other cultivars. The results of this study indicate that Sudanese date extracts have an intermediate H_2_O_2_ scavenging capacity. In agreement with our results, a concentration-dependent H_2_O_2_ scavenging ability has been reported for palm date syrups from various countries (Al-Mamary et al. 2010). Moreover, low to high values of H_2_O_2_ scavenging capacity for Yemeni, Saudi, and Iraqi date syrups (Al-Mamary et al. 2010) and for Algerian dates (Benmeddour et al. 2013) have been reported. H_2_O_2_ is a weak oxidizing agent and can inactivate a few enzymes directly by oxidation of essential thiol (-SH) groups. However, the H_2_O_2_ can penetrate cell membranes rapidly. Once inside the cell, it may react with Fe^2+^ and possibly Cu^2+^ ions to form hydroxyl radicals and this could be the source of its toxicity. Thus, it is important for cells to avoid an accumulation of H_2_O_2_. Therefore, consuming diets with high H_2_O_2_ scavenging capacity such as date fruits is highly recommended because this could possibly reduce and/or abolish the formation of H_2_O_2_, and hence save the body from oxidative damage.

### Correlation between antioxidant activity and mineral extractability

The results in Table 6 present the correlations between antioxidants and mineral extractability of Sudanese date fruit extracts. Generally, our results showed that there were many correlations (positive, negative, weak) between antioxidant and mineral extractability. Significantly positive correlations were observed between total flavonoids and extractability of both macro- and microminerals in date fruit extracts. Total flavonoid content showed extremely positive correlations (**P < 0.01) with K (*r*^2^ = 0.889) and Mg (*r*^2^ = 0.776) and significantly positive correlations (*P < 0.05) with Mn (*r*^2^ = 0.485) as well as significantly (*P < 0.05) negative correlation with Ca (*r*^2^ = −0.588). These findings suggest interaction between total flavonoids and mineral extractability, which could result in either reduction or enhancement of the bioavailability of these minerals. On the other hand, TPC was positively correlated (**P < 0.01) with Na (*r*^2^ = 0.826) and P (*r*^2^ = 0.598), but negatively correlated (**P < 0.01) with Zn (*r*^2^ = −0.851). These results demonstrate that total polyphenol in date fruits has a significant impact on enhancing the extractability of both sodium and phosphorus, but they have no effect on the extractability of zinc. Moreover, these results are related with the ability to chelate ion metals by polyphenol compounds and to retain more effectively the complex into the macromolecular structure formed by condensed flavonoids as reported for extracts of some medicinal plants (Weber and Konieczynski 2003; Whittaker et al. 2009). Thus, the chelating role of these metals observed in Sudanese date fruit extracts could have potential interest as a dietary antioxidant in the food industry, preventing or delaying metal-catalyzed initiation and decomposition of lipid hydroperoxides.

**Table 6:** Correlation coefficient of antioxidant activity and mineral extractability of Sudanese dates varieties

Pearson correlation	Ca	Mg	Na	K	P	Fe	Cu	Zn	Mn
Total flavonoid content (TFC)	−0.588*	0.776**	−0.191	0.889**	−0.399	0.073	0.173	−0.061	0.485*
Total polyphenol content (TPC)	0.103	−0.105	0.826**	−0.078	0.598**	0.302	−0.467	−0.851**	−0.370
Ferric reducing power (FRAP125)	0.496*	−0.402	0.068	0.800**	−0.507*	−0.281	−0.121	−0.616**	−0.443
Chelation of Fe^2+^ (Ch125)	0.309	0.610**	−0.830**	−0.020	−0.161	−0.037	0.015	0.753**	−0.018
Scavenging of H_2_O_2_ (SC125)	−0.789**	0.045	−0.110	0.726**	−0.534*	−0.094	−0.332	−0.263	0.575*
FRAP250	0.176	−0.025	0.213	0.337	−0.262	−0.254	−0.425	−0.745**	−0.078
Ch250	0.209	0.666**	−0.808**	−0.100	−0.230	−0.077	0.109	0.729**	−0.096
SC250	−0.055	0.258	−0.127	0.233	−0.171	−0.404	−0.786**	−0.574*	0.367
FRAP500	0.088	−0.135	0.283	0.065	−0.073	−0.287	−0.505*	−0.745**	−0.092
Ch500	0.245	0.385	−0.856**	−0.061	−0.565*	−0.581*	0.158	0.588*	−0.284
SC500	−0.191	0.014	−0.717**	0.178	−0.647**	−0.257	−0.104	−0.467	0.260
FRAP1000	0.664**	−0.226	0.026	0.559*	−0.244	−0.189	−0.589*	−0.202	−0.488*
Ch1000	0.160	0.093	−0.436	−0.123	−0.583*	−0.728**	0.125	0.075	−0.253
SC1000	−0.065	0.210	−0.697**	0.086	−0.377	−0.034	−0.421	−0.247	0.110

* Correlation is significant at *P* < 0.05.

** Correlation is significant at *P* < 0.01.

In addition, a high correlation between antioxidant activities of date fruit extracts and mineral extractability was also observed (Table 6). The FRAP of date fruits extracted at different concentrations (125–1000 *μ*g/mL) showed a highly significant (**P < 0.01) positive correlation with K (*r*^2^ = 0.800) and Ca (*r*^2^ = 0.664) but a highly significant (**P < 0.01) negative correlation with Zn (*r*^2^ = −0.745) and significant (*P < 0.05) negative correlation with Cu (*r*^2^ = −0.589), and Mn (*r*^2^ = −0.488). Moreover, chelation of Fe^2+^ of date fruit extracts at various concentrations correlated positively (**P < 0.01) with Mg (*r*^2^ = 0.666) and Zn (*r*^2^ = 0.753), but showed a major significantly negative (**P < 0.01) correlation with Na (*r*^2^ = −0.856) and Fe (*r*^2^ = −0.728), and a significantly negative (*P < 0.05) correlation with P (*r*^2^ = −0.728). The results also showed no correlation between iron chelating activity and potassium and copper extractability. Additionally, scavenging of H_2_O_2_ of Sudanese dates extracts was positively correlated with K (**P < 0.01, *r*^2^ = 0.726) and with Mn (*P < 0.05, *r*^2^ = 0.575) and negatively (**P < 0.01) correlated with Ca (*r*^2^ = −0.789), Na (*r*^2^ = −0.717), P (*r*^2^ = −0.647), and Cu (*r*^2^ = −0.786). It is worth noting that there was a highly significant (**P < 0.01) and significant (*P < 0.05) correlation between antioxidant capacity and mineral extractability of date palm fruit extracts. Despite the wealth of literature available on minerals and antioxidant content of date fruits, there are unfortunately hardly any available studies on the correlation between mineral extractability, polyphenol and flavonoid content, and antioxidant activity of dates. Thus, this is the first report on the correlation between antioxidant capacity and mineral extractability not only on date fruits but also on other fruits and vegetables. All previously published reports described the correlation of polyphenols and antioxidants with mineral content but not with mineral extractability (Sulaiman et al. 2011; Perna et al. 2012). It would be interesting to analyze the correlation between mineral extractability and isolated antioxidant phenolic and flavonoids compounds with the aim of establishing a more precise correlation between antioxidants and mineral extractability of date fruits.

## Conclusions

This study reported on the total flavonoid and polyphenol content, mineral content and extractability, and antioxidant capacities of Sudanese date fruits for the first time. Moreover, the correlation between antioxidant capacity and mineral extractability of date fruits was also attempted for the first time. Sudanese date varieties demonstrated high amounts of macro- and micromineral content with a significant varietal dependence. Generally, lower extractability was observed in most of the macrominerals and some microminerals. Regarding antioxidant constituents, Sudanese date varieties have high amounts of total polyphenols and total flavonoids, suggesting potential protection capabilities of these dates against the action of reactive oxygen species. Moreover, the FRAP, chelation of Fe^2+^ ion and H_2_O_2_ scavenging of Sudanese date fruits demonstrated moderate to high values and varied significantly between varieties. Strikingly, the correlations of antioxidants, total polyphenols, and flavonoids with mineral extractability suggested the influence of antioxidant compounds on mineral bioavailability.
